# Reproducibility of quantitative myocardial perfusion and coronary flow capacity by positron emission tomography: 3D digital silicon photomultiplier solid state vs. legacy 2D analogue systems for clinical practice and trials

**DOI:** 10.1093/ehjimp/qyae115

**Published:** 2024-12-09

**Authors:** Amanda Roby, Lindsey Harmon, Kelly Sander, Linh Bui, Danai Kitkungvan, Monica Patel, Jagat Narula, Nils P Johnson, K Lance Gould

**Affiliations:** Weatherhead PET Center for Preventing and Reversing Atherosclerosis, Division of Cardiology, Department of Medicine, McGovern Medical School, University of Texas Health Science Center at Houston, Memorial Hermann Hospital, 6431 Fannin St., Room MSB 4.256 Houston, TX 77030, USA; Weatherhead PET Center for Preventing and Reversing Atherosclerosis, Division of Cardiology, Department of Medicine, McGovern Medical School, University of Texas Health Science Center at Houston, Memorial Hermann Hospital, 6431 Fannin St., Room MSB 4.256 Houston, TX 77030, USA; Weatherhead PET Center for Preventing and Reversing Atherosclerosis, Division of Cardiology, Department of Medicine, McGovern Medical School, University of Texas Health Science Center at Houston, Memorial Hermann Hospital, 6431 Fannin St., Room MSB 4.256 Houston, TX 77030, USA; Weatherhead PET Center for Preventing and Reversing Atherosclerosis, Division of Cardiology, Department of Medicine, McGovern Medical School, University of Texas Health Science Center at Houston, Memorial Hermann Hospital, 6431 Fannin St., Room MSB 4.256 Houston, TX 77030, USA; Weatherhead PET Center for Preventing and Reversing Atherosclerosis, Division of Cardiology, Department of Medicine, McGovern Medical School, University of Texas Health Science Center at Houston, Memorial Hermann Hospital, 6431 Fannin St., Room MSB 4.256 Houston, TX 77030, USA; Weatherhead PET Center for Preventing and Reversing Atherosclerosis, Division of Cardiology, Department of Medicine, McGovern Medical School, University of Texas Health Science Center at Houston, Memorial Hermann Hospital, 6431 Fannin St., Room MSB 4.256 Houston, TX 77030, USA; Weatherhead PET Center for Preventing and Reversing Atherosclerosis, Division of Cardiology, Department of Medicine, McGovern Medical School, University of Texas Health Science Center at Houston, Memorial Hermann Hospital, 6431 Fannin St., Room MSB 4.256 Houston, TX 77030, USA; Weatherhead PET Center for Preventing and Reversing Atherosclerosis, Division of Cardiology, Department of Medicine, McGovern Medical School, University of Texas Health Science Center at Houston, Memorial Hermann Hospital, 6431 Fannin St., Room MSB 4.256 Houston, TX 77030, USA; Weatherhead PET Center for Preventing and Reversing Atherosclerosis, Division of Cardiology, Department of Medicine, McGovern Medical School, University of Texas Health Science Center at Houston, Memorial Hermann Hospital, 6431 Fannin St., Room MSB 4.256 Houston, TX 77030, USA

**Keywords:** coronary physiology, quantitative myocardial perfusion, coronary flow capacity, coronary flow reserve, positron emission tomography, myocardial blood flow

## Abstract

**Aims:**

Quantitative rest–stress myocardial perfusion in millilitres per minute per gram among multiple 2D and 3D positron emission tomography–computed tomography (PET-CT) scanners is essential for personalized cardiac management and clinical trials. Accordingly, this study reports the accuracy and precision of quantitative rest–stress millilitres per minute per gram and coronary flow capacity among 2D and two different digital 3D silicon photomultiplier (SiPM) PET-CT scanners for quantifying the severity of coronary pathophysiology for clinical trials or guiding interventions vs. medical treatment.

**Methods and results:**

One hundred seventy-one participants underwent 748 paired serial rest or stress PET perfusion imaging in the same person on ‘same day’ or ‘different days’ using rubidium-82 (Rb-82) pharmacologic stress on 2D and two different digital 3D SiPM PET-CT scanners for global myocardial perfusion in millilitres per minute per gram. For methodological variability of 66 ‘same-day’ serial paired PETs in the same person by 2D and two different 3D SiPM PET-CT scanners, rest–stress global myocardial millilitres per minute per gram had no significant bias (*P* = 0.464, mean difference 0.014 ± 0.21 mL/min/g) with coefficient of variation (COV) of ±14%. For methodological plus biological variability of 154 ‘different-day’ serial paired PETs, rest–stress global perfusion had no significant bias (*P* = 0.136), mean difference (0.028 ± 0.33), and COV of ±20%. Coronary flow reserve had a small bias of 0.095 ± 0.57 (*P* = 0.041) and COV of ±20%. Coronary flow capacity was not different by Kolmogorov–Smirnov test (*P* = 0.99).

**Conclusion:**

For quantifying myocardial perfusion in the same person on ‘same day’ or ‘different days’ using Rb-82, 3D SiPM PET-CT is comparably reproducible to analogue 2D PET-CT with the HeartSee perfusion model as the basis for quantifying physiologic severity of coronary heart disease to guide clinical decision-making or randomized clinical trials confirming these outcomes.

## Structured Graphical Abstract

**Structured Graphical Abstract:**
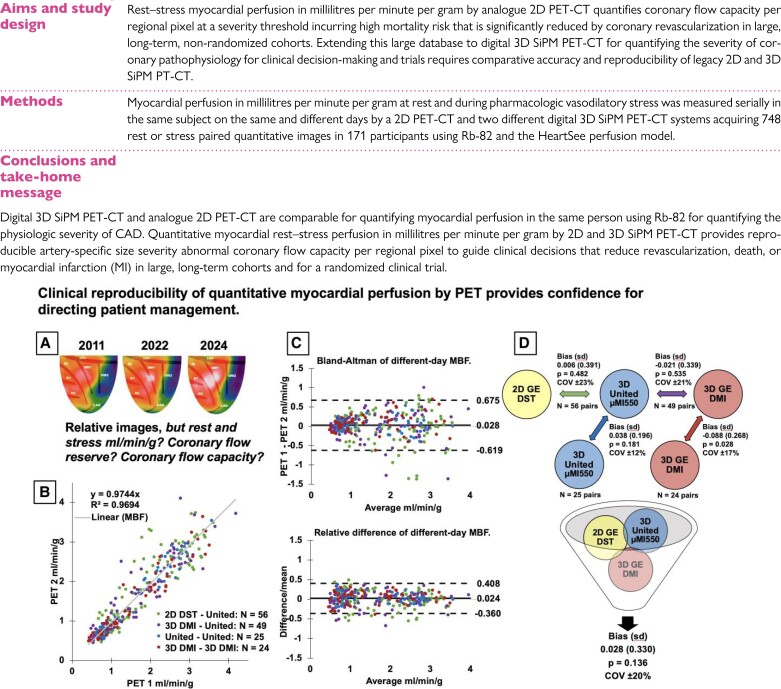
Rationale and summary of the study. In large non-randomized patient cohorts and in a randomized trial followed over 14 years, CFC by 2D PET-CT quantifies CAD severity favouring lifestyle-medical treatment while reducing coronary interventions to physiologically severe, high-risk, obstructive CAD having survival benefit after revascularization. Documenting equivalency, reproducibility, and precision of high-sensitivity 3D SiPM PET-CT compared with 2D PET-CT systems extends this extensive knowledge base derived from 2D PET-CT using Rb-82. The addition of 3D SiPM PET-CT with optimal acquisition–reconstruction protocols and perfusion models provides confidence to physicians and patients in quantifying physiologic CAD severity to guide personalized management and randomized trials of CAD management. For a patient with serial scans over 13 years (*A*), relative stress images are unchanged on 2D GE DST, 3D United µMI550, and 3D GE DMI PET-CT without quantitative perfusion defining the status of severe CAD. Subgroup perfusion correlation in millilitres per minute per gram (*B*) among all perfusion measurements with Bland–Altman plots (*C*) of perfusion difference and relative difference (difference/mean) is the evidence for their combined comparison (*D*). Serial 154 rest–stress millilitres per minute per gram paired measurements on different days in the same person between analogue 2D GE DST, the 3D United µMI550, and the 3D GE DMI PET-CT systems produce the largest comparison to date of ‘different-day’ perfusion (*B*, *C*, *D*). Mean COV is ±20% for methodological plus day-to-different-day biological variability, providing confidence to physicians and patients for quantifying physiologic CAD severity to guide personalized management or randomized trials (*D*).

## Introduction

In large non-randomized patient cohorts followed over 14 years, coronary flow capacity (CFC) by 2D PET-CT quantifies CAD severity favouring lifestyle-medical treatment while reducing coronary interventions to physiologically severe, high-risk, obstructive CAD having survival benefit after revascularization in large, long-term, non-randomized cohorts.^1–4^ Documenting equivalency, reproducibility, and precision of high-sensitivity 3D silicon photomultiplier (SiPM) positron emission tomography–computed tomography (PET-CT) compared with 2D PET-CT systems extends this knowledge base derived from earlier versions of 2D PET-CT scanners, thereby providing confidence to physicians and patients in quantifying physiologic CAD severity to guide clinical practice and trials.

3D mode imaging for PET has introduced numerous improvements and challenges for quantifying myocardial perfusion. However, a study of 10 analogue 3D PET-CT scanners revealed substantial variability within models and vendors requiring limited dose ranges for quantitative myocardial perfusion with rubidium-82 (Rb-82).^5^ Recent digital PET detector technology merges lutetium-based scintillator crystals and SiPM blocks, improving scintillation kinetics (rise and decay times) and coincidence time resolution for quantifying high-count arterial input and myocardial perfusion.^6^

The literature survey summarized in *Table 1* for ‘different-day’ comparisons shows that rest–stress millilitres per minute per gram and coronary flow reserve (CFR) have not been validated for test–retest variability of current digital 3D SiPM PET-CT compared with analogue 2D PET-CT or for serial measurements on the same 3D SiPM PET-CT. The limited clinical literature on 3D SiPM PET-CT suggests adequate and reproducible ‘different-day’ perfusion measurements when time-activity curves were adequate that, however, were inconsistent in 20% for unclear reasons.^7^ For legacy analogue 2D PET-CT, we previously compared the accuracy and precision of ‘same-day’ and ‘different-day’ quantitative perfusion and CFC in paired serial PETs in the same patient.^8^ Other recent concerns for quantifying perfusion by PET have focused on the impact of motion, low perfusion in transmural infarct, disagreement between commercially available packages, and significant proportion (20%) with inadequate time-activity curves precluding perfusion measurements.^9–11^ ‘Same-day’ comparisons share similar issues (see Supplementary data online, *Table S0*).

**Table 1:** Review of published literature examining different-day test–retest of PET MBF

Author	Year	*n*	2D/3D/3D SiPM	Tracer	Rest bias ± SD or (95% CI)	*t*-test *P*	COV (±%)	Stress bias ± SD or (95% CI)	*t*-test *P*	COV (±%)	CFR bias ± SD or (95% CI)	*t*-test *P*	COV (±%)
Nagamachi^S1^	1996	13	2D	N-13	11.5% ± 12.2%	<0.05		11.4% ± 11.5%	ns				
Jagathesan^S2^	2005	15	2D	O-15	0.08 ± 0.13	ns	12	0.07 ± 0.3	ns	15	0.08 ± 0.33	ns	17
Schindler^S3^	2007	20	2D	O-15	0.1 ± 0.1	ns	17	0.14 ± 0.1	ns	12			
Sdringola^S4^	2011	59	2D	Rb-82	0.01 ± 0.12	ns	17	0.18 ± 0.55	<0.05	21	0.2 ± 1.00	ns	26
Sdringola^S4^	2011	48	2D	Rb-82	0.05 ± 0.13	<0.05	18	0.01 ± 0.5	ns	17	−0.22 ± 0.81	ns	19
Johnson^S5^	2015	50	2D	Rb-82	−0.02 ± 0.17	0.46		−0.09 ± 0.39	0.13		−0.07 ± 0.48	0.29	
Kitkungvan^S6^	2017	19	2D	Rb-82						17			20
Kitkungvan^S7^	2017	120	2D	Rb-82	0.07 ± 0.2	0.13	21	0.02 ± 0.46	0.81	19			
Koenders^S8^	2020	30	3D/3D SiPM	Rb-82	*n* = 28	≥0.29	≤21	*n* = 25	≥0.11	≤21	*n* = 24	≥0.51	≤21
Manabe^S9^	2020	19	2D/3D	Rb-82		0.74			0.84			0.66	
Byrne^S10^	2021	36	3D syngo.MBF	Rb-82	−0.04 (−0.1 to 0.03)	0.25		0.05 (−0.18 to 0.28)	0.67		0.2 (−0.07 to 0.47)	0.13	23
Byrne^S10^	2021	36	3D QGS	Rb-82	−0.04 (−0.1 to 0.19)	0.18		−0.03 (−0.2 to 0.15)	0.76		0.17 (−0.1 to 0.44)	0.21	23
Byrne^S10^	2021	36	3D 4DM	Rb-82	0.02 (−0.05 to 0.08)	0.63		0.03 (−0.18 to 0.23)	0.81		0.08 (−0.17 to 0.34)	0.5	20
Byrne^S10^	2021	36	3D 4DM MC	Rb-82	−0.05 (−0.1 to 0.01)	0.09		−0.04 (−0.25 to 0.18)	0.72		0.17 (−0.17 to 0.51)	0.32	27
Current study		56	2D/3D SiPM	Rb-82	0.060 ± 0.219	0.039	23	−0.050 ± 0.504	0.500	20	−0.283 ± 0.581	0.001	21
Current study		49	3D SiPM	Rb-82	0.011 ± 0.204	0.707	23	0.043 ± 0.394	0.450	17	0.081 ± 0.513	0.275	18
Current study		24	3D SiPM	Rb-82	0.061 ± 0.152	0.063	17	0.117 ± 0.349	0.115	15	0.028 ± 0.556	0.808	19
Current study		25	3D SiPM	Rb-82	0.073 ± 0.130	0.010	15	0.003 ± 0.244	0.955	10	−0.236 ± 0.532	0.036	18
Current study		154	2D/3D SiPM	Rb-82	0.045 ± 0.194	0.005	22	0.011 ± 0.425	0.745	18	0.095 ± 0.571	0.041	20

Superscript numbers as S1, S2, S3, S4, S5, S6, S7, S8, S9, and S10 refer to the references in the Supplementary data.

Accordingly, we used the ‘simple retention’ perfusion model based on time integration to improve pixel statistics, reduce motion artefact, with instrument-specific partial volume correction, optimal patient and phase-specific arterial input selection, and avoid rigid arbitrary segmentation.^12^ This acquisition–perfusion model is validated experimentally^12^ in normal volunteers^13^ for reproducibility,^8^ including myocardial infarcts,^10,11^ by angina and ST depression > 1 mm during PET stress^14^ and by clinical outcomes.^1–4,14,15^

## Methods

Quantitative myocardial perfusion was measured by serial paired PET-CT in the same person for comparative accuracy and reproducibility between analogue 2D and two different digital 3D SiPM PET-CT systems at the Weatherhead PET Center for Preventing and Reversing Atherosclerosis, McGovern Medical School, University of Texas Health Science Center at Houston.

### Subjects

Participants received serial PET exams within radiation safety guidelines, totalling 748 PET acquisitions from 171 participants recruited by referral from UT clinics. Exclusion criteria included absolute contraindication to dipyridamole, pregnancy or active breastfeeding, current participation in other clinical research, and inability to undergo two PET scans on same day or within 1–8 weeks apart and to abstain from caffeine for 24 h prior to imaging.

**Figure 1:**
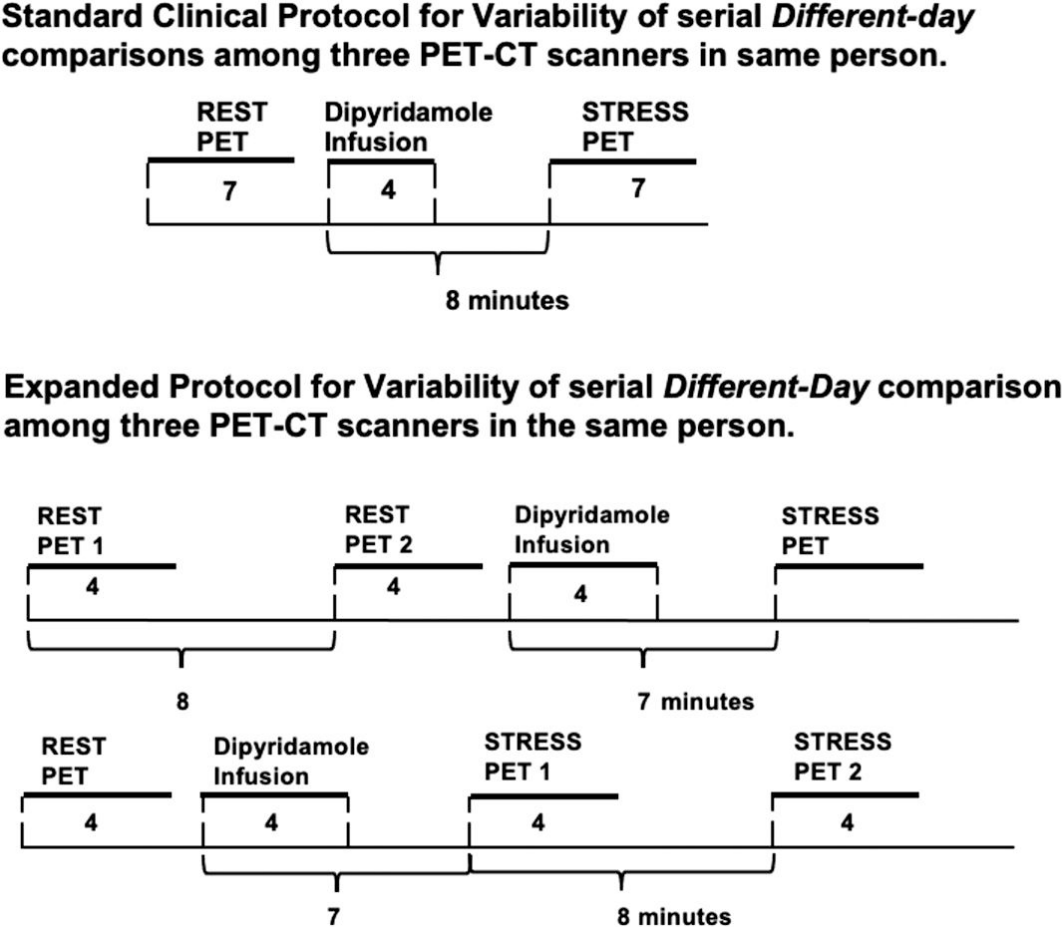
Protocol for comparing ‘same-day’ and ‘different-day’ serial rest–stress millilitres per minute per gram, CFR, and CFC in the same subject.

Standard imaging protocols were used as previously reported (*Figure 1*).^1–4,8,13–21^ For methodological variability without ‘day-to-different-day’ biological variability, rest myocardial perfusion in millilitres per minute per gram was measured by serial ‘same-day’ pairs in the same patient between 2D and 3D SiPM PET-CT and in rest–stress ‘same-day’ pairs comparing same 3D SiPM PET-CT system to itself (*Figure 1*). For cumulative ‘day-to-different-day’ (biological plus methodological) variability, rest–stress myocardial perfusion was measured on serial ‘different-day’ pairs in the same patient among three PET-CT systems. Participants were randomly assigned using www.sealedenvelope.com to one protocol on Day 1 alternating with Day 2.

### Cardiac PET acquisition and analysis

Cardiac PET was performed on three different PET-CT systems: 16-slice BGO analogue PET-CT scanner (Discovery DST, GE Healthcare, Waukesha, WI, USA) in 2D mode; digital 3D PET-CT (United Imaging µMI550 SiPM, Houston, TX, USA) with 20 mm CT coverage; and digital 3D, 4-ring SiPM 64-slice PET-CT, (GE DMI, GE Healthcare, Waukesha, WI, USA). All myocardial perfusion analyses were performed employing FDA-approved 510K-231731 HeartSee software (or Bracco Diagnostics, Inc., NJ, USA).

Attenuation correction was acquired by reduced dose computed tomography using both cine and helical acquisitions on all three systems, one before rest and the other after stress; PET and CT data were co-registered by manually shifting CT data to fit Rb-82 myocardial uptake data and reconstructed as previously reported.^1–4,8,13–21^

Acquiring quantitatively accurate high-count myocardial perfusion on 3D PET systems is complexly different from 2D systems, particularly for rapidly changing, high-activity arterial input function with Rb-82, which has the potential to degrade arterial activity recovery and falsely lower arterial input values.^5–7,17,19,21^

All PET acquisitions were acquired in list mode and reconstructed as similarly as brand differences allow. Protocols are structured to acquire high-count rubidium activity accurately corrected for random coincidences, scatter, and dead time loss that are essential for quantifying myocardial tissue in millilitres per minute per gram. 2D DST PET acquisition is performed dynamically as two frames: a 2-min arterial input and a 5-min relative perfusion, reconstructed via filtered back projection with Butterworth order 10 and a cut-off of 15 mm and then split into two static images for post-processing.

United hardware and software dynamically correct PET list mode data for scatter, randoms, dead time, singles, prompt gamma, and decay during acquisition for static reconstructions of arterial input and relative perfusion images. United PET acquisitions were reconstructed with time of flight (TOF), point spread function (PSF), ordered subset expectation maximization (OSEM) of two iterations, and 20 subsets with an added Gaussian smoothing filter ‘Smooth 3’ with a full-width half maximum of 7 mm.

The GE DMI protocol is designed to compare with United as closely as software, hardware, and clinical efficiency allow. 3D DMI list mode applies data corrections based on acquisition time structure, requiring short dynamic frame timing during the first pass to ensure accurate quantification of reconstructed images per study subgroup into 34- or 28-time frames (24 × 5 s + 10 × 30 s or 24 × 5 s + 4 × 30 s) and summing in GE Dynamic VUE software (GE Healthcare) to produce a single static arterial input and relative perfusion image. 3D DMI used TOF, PSF, and OSEM of two iterations and 34 subsets, with Butterworth order 10 and cut-off of 15 mm.

Rest and stress data were acquired with intravenous injection of 1100–1850 kBq (30–50 mCi) at 50 mL/min of generator-produced Rb-82 (Bracco Diagnostics, Princeton, NJ, USA). Acquisitions started when the generator switched from waste to patient infusion.

All studies used pharmacologic stress of either adenosine or dipyridamole. As previously reported, ‘same-day’ test–retest precision of stress perfusion in millilitres per minute per gram requires dipyridamole for sustained hyperaemia of approximately 15 min^8^ with a delay of 6.3 half-lives between Rb-82 infusions to decay residual activity. Hence, all same-day stress comparisons used dipyridamole. Blood caffeine was measured each study day.^20^

**Figure 2:**
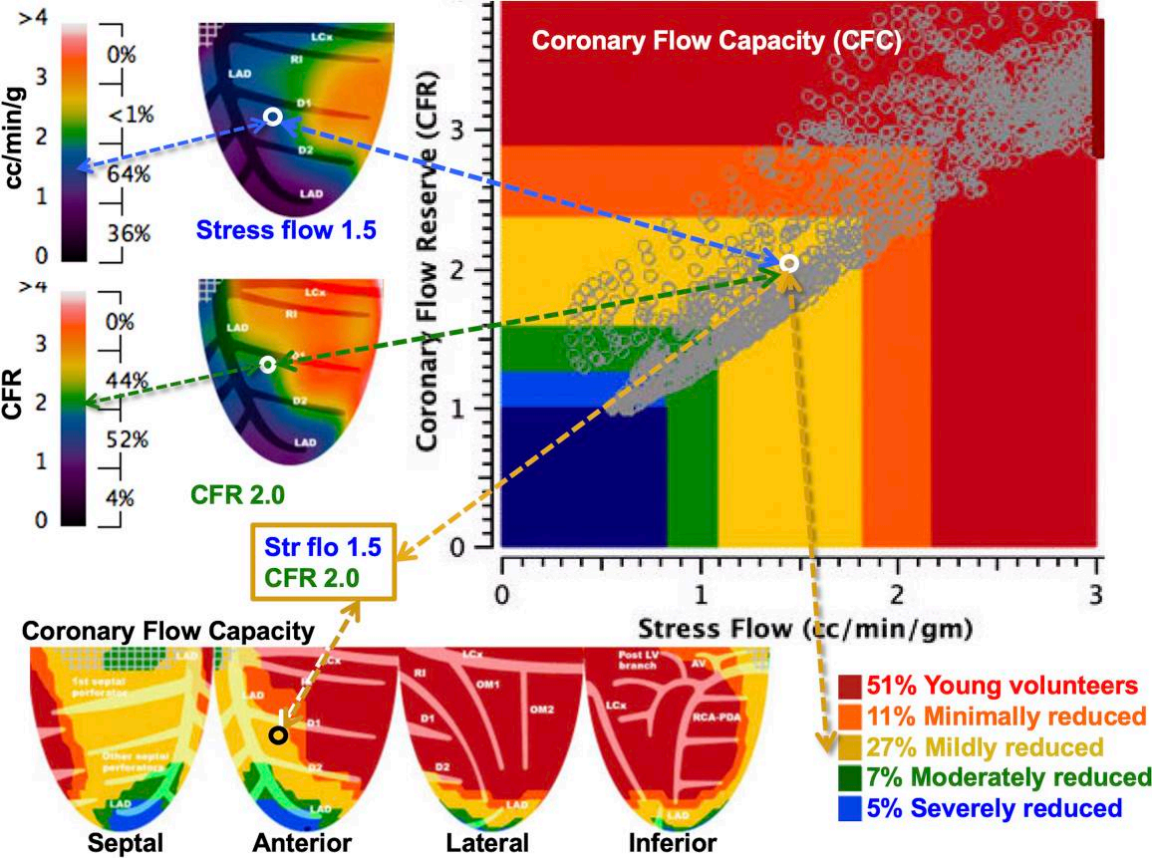
CFC map objectively quantifies rest–stress perfusion and CFR per regional pixel and their combination in pre-specified ranges, colour coded by well-defined clinical groups and back projected into their LV position. Artery-specific size severity of CFC as a percentage of LV is the comprehensive, integrated perfusion metric associated with risk of adverse events with and without revascularization (see text).

Global perfusion values are averages of the total 1344 pixels of left ventricle (LV). Quadrant perfusion values are averages of 336 non-overlapping pixels in septal, anterior, lateral, and inferior quadrant views. Absolute myocardial perfusion, in millilitres per minute per gram per pixel, is quantified by separate optimized arterial input locations for rest and stress.^16,17^ CFR is computed as a stress-to-rest ratio per pixel. CFC maps plot each pixel value of stress perfusion and CFR to calculate the CFC as percentage of LV plotted within previously established patient-driven ranges as in *Figure 2*.^1–4,8,13–21^ Pixel-level, artery-specific distribution provides severity, size, and regional distribution of rest–stress perfusion abnormalities without arbitrary externally imposed regions of interest (ROIs) that commonly include overlapping coronary arterial distributions.^1–4,8,13–21^

Scanner-specific partial volume corrections were determined by phantom testing and optimized in software for all three PET-CT systems.^17,19,21^ All perfusion metrics were objectively made by automated software by two of three experienced, highly trained cardiac PET technologists. Two experienced cardiologists highly trained in coronary pathophysiology, cardiac PET technology, and clinical cardiology made a final cross-check on technical aspects and clinical interpretation of every PET and then checked in detail by the senior author, all blinded to any prior PET before fixing the final objective automated perfusion metrics.

### Statistical analysis

Global, quadrant, pixel values of rest, stress, CFR, and CFC determined precision and variability using R 4.3.1 (R Foundation for Statistical Computing, Vienna, Austria), and standard summary statistical tests were used for analysis. Linear regression is reported between scanners, rest perfusion, and BMI and for combined subgroups. Applicable tests are two tailed, and *P* < 0.05 is considered statistically significant. Student’s *t*-tests evaluate continuous variables where appropriate. The Pitman–Morgan *F*-test is used to test for differences in the variability between test groups. A Kolmogorov–Smirnov test for differences in histogram distributions is used to compare colour-coded ranges of CFC maps.^1–4,8,13–21^

## Results

### Study population

One hundred eighty-one participants consented to the study. Seven stress scans (4%) were excluded due to measured blood caffeine, and three (1.6%) exams due to patient withdrawal or initial scans discovered disease that required urgent revascularization, thereby removing them from the protocol leaving 171 participants undergoing 748 paired rest or stress PET-CT scans paired in the same person. Consequently, data were analysed for 154 rest–stress pairs (*Table 2*) and separately 17 rest-only pairs (see Supplementary data online, *Figure S1*). No technical scanner failures precluded PET data evaluation. No (zero) cases were excluded due to poor arterial bolus or motion invalidating accurate arterial input calculation or failure to yield perfusion metrics during post-processing.

**Table 2:** Participant demographics by subgroup

Characteristic	Overall^a^	2D DST—United^a^	3D DMI—United^a^	3D DMI—3D DMI^a^	United—United^a^
	*n* = 154	*n* = 56	*n* = 49	*n* = 24	*n* = 25
Male	99/154 (64%)	37/56 (66%)	32/49 (65%)	17/24 (71%)	13/25 (52%)
Age	58 (12)	56 (15)	60 (9)	59 (9)	56 (11)
BMI	29 (6)	29 (6)	29 (6)	29 (3)	29 (5)
Stress agent					
Adenosine	4/154 (3%)	0/56 (0%)	4/49 (8.2%)	0/24 (0%)	0/25 (0%)
Dipyridamole	150/154 (97%)	56/56 (100%)	45/49 (94%)	24/24 (100%)	25/25 (100%)
PET angina	2/154 (1%)	1/56 (1.8%)	0/49 (0%)	0/24 (0%)	1/25 (4.0%)
PET ST depression > 1 mm	7/154 (5%)	5/56 (8.9%)	1/49 (2.1%)	0/24 (0%)	1/25 (4.0%)
Prior PCI	21/154 (14%)	12/56 (21%)	7/49 (14.3%)	1/24 (4.2%)	1/25 (4.0%)
Prior CABG	6/154 (4%)	5/56 (8.9%)	0/49 (0%)	0/24 (0%)	1/25 (4.0%)
History of MI	12/154 (12%)	8/56 (14%)	3/49 (6.3%)	0/24 (0%)	1/25 (4.0%)
Any coronary calcium	99/154 (64%)	41/56 (73%)	31/49 (63%)	17/24 (71%)	10/25 (40%)
History of hypertension	85/154 (55%)	29/56 (52%)	33/49 (67%)	13/24 (54%)	10/25 (40%)
History of dyslipidaemia	88/154 (57%)	33/56 (59%)	32/49 (65%)	13/24 (54%)	10/25 (40%)
History of diabetes	40/154 (26%)	15/56 (27%)	15/49 (31%)	5/24 (21%)	5/25 (20%)
History of smoking					
Non-smoker	119/154 (77%)	43/56 (77%)	36/49 (75%)	16/24 (67%)	24/25 (96%)
Quit	11/154 (7%)	8/56 (14%)	1/49 (2.1%)	2/24 (8.3%)	0/25 (0%)
Smoker	23/154 (15%)	5/56 (8.9%)	11/49 (23%)	6/24 (25%)	1/25 (4.0%)

CABG, coronary artery bypass surgery; MI, myocardial infarction.

^a^ *n*/*n* (%); mean (SD).

Of 154 serial PET pairs, 27% were abnormal with CFC severe (blue) or CFC moderate (green) > 1% of LV or CFC mild (yellow) > 10% of LV. Of 154 PET pairs, four (2.6%) had sufficient differences to report with two worse and two better, three ascribable to 3D count density and resolution compared with 2D, and one due to hypotension of hypovolaemia. One of the three, or one of 154, had definite abnormal regional quantitative CFC on 2D PET-CT that was not severe enough to recommend percutaneous coronary intervention (PCI) but was sufficiently worse on 3D PET-CT to recommend PCI.

### Methodological variability by same-day serial test–retest rest and stress myocardial perfusion in the same subject by three PET-CT scanners

**Figure 3:**
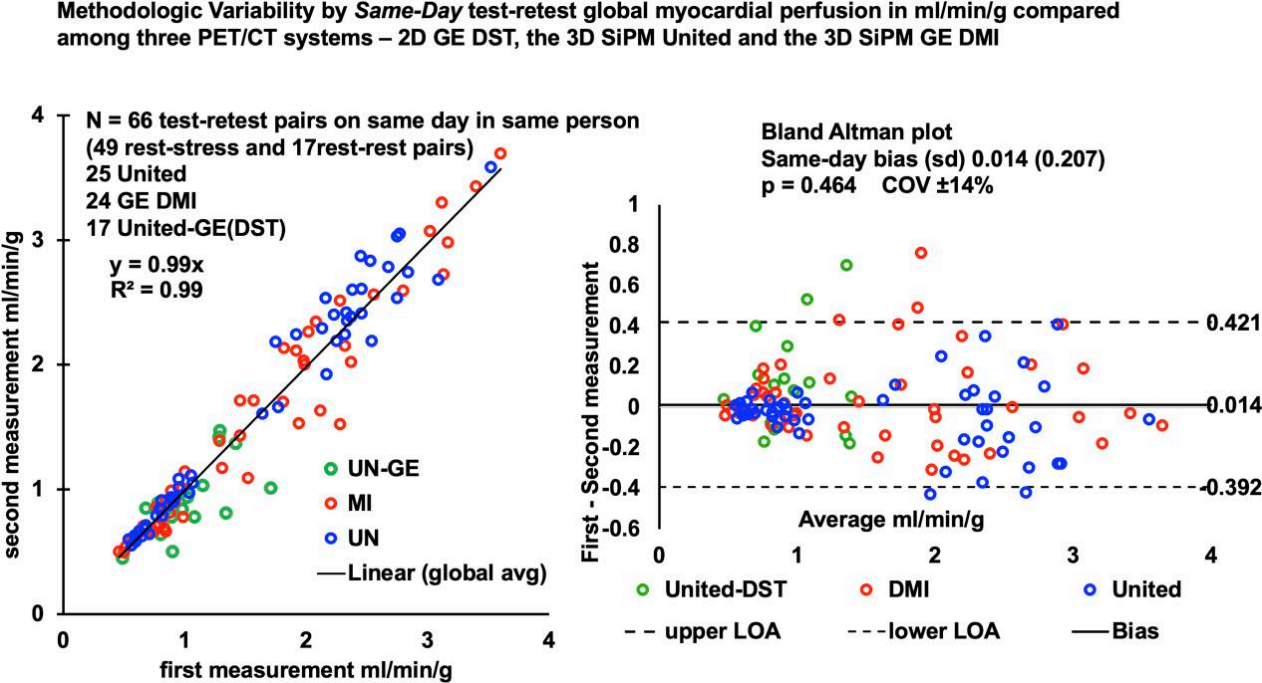
Methodological variability of serial paired ‘same-day’ global rest–stress myocardial perfusion in millilitres per minute per gram in the same subject between 2D GE DST and 3D United µMI550 and the 3D United µMI550 and 3D GE DMI PET-CT scanners compared with themselves with a combined COV of ±14%, in contrast to methodological (same-day) plus biologic (different-day) variability of ±20% from *Figure 5* (*Graphical Abstract*).

To assess methodological variability among the three PET-CT scanners without ‘day-to-different-day’ biological variability, we performed serial paired ‘same-day’ rest–rest and rest–stress myocardial perfusion in 66 clinical referral participants (*Table 3*). No scanner suggested saturation by a reduction of counts during maximal arterial phase activity. *Figure 3* summarizes the linear relationship of myocardial perfusion in millilitres per minute per gram and Bland–Altman plot describes the similarity of 66 ‘same-day’ paired serial PETs acquired on all three PET-CT scanners with no significant difference between all paired measurements (*r* = 0.99) with a bias of 0.014 ± 0.21 (paired *P* = 0.464, Pitman–Morgan *P* = 0.401) and a coefficient of variation (COV) of ±14%, consistent with our previous report of ‘same-day’ variability^4^ and prior publications (*Table 1*). While ‘same-day’ methodological variability reported here is essential for comparing all three 2D and 3D PET-CT scanner functions without biological variability, day-to-different-day biological plus methodological variability is most relevant for clinical use.

**Table 3:** Global absolute flow millilitres per minute per gram comparisons

	*n* pairs	Difference (SD)	Paired *t*-test *P*-value	Pitman–Morgan *P*-value	COV
**Different day**					
**Different scanner**					
2D DST—United	56	0.006 (0.391)	0.482	0.321	±23%
United—3D DMI	49	−0.021 (0.339)	0.535	0.476	±21%
**Same scanner**					
3D DMI—3D DMI	24	−0.088 (0.268)	0.028	0.334	±17%
United—United	25	0.038 (0.196)	0.181	0.622	±12%
**Different-day combined comparison**	**154**	**0.028** (**0.330)**	**0**.**136**	**0**.**419**	**±20%**
**Same day**					
**Different scanner**					
2D DST—United	17	−0.111 (0.250)	0.087	0.375	±26%
**Same scanner**					
3D DMI—3D DMI	24	0.039 (0.214)	0.213	0.492	±14%
United—United	25	−0.050 (0.178)	0.053	0.436	±11%
**Same-day combined comparison**	**66**	**0.014** (**0.207)**	**0**.**464**	**0**.**401**	**±14%**

### Biological plus methodological variability by different-day serial test–retest rest–stress myocardial perfusion in the same subject for 2D DST vs. 3D µMI550 PET-CT

**Figure 4:**
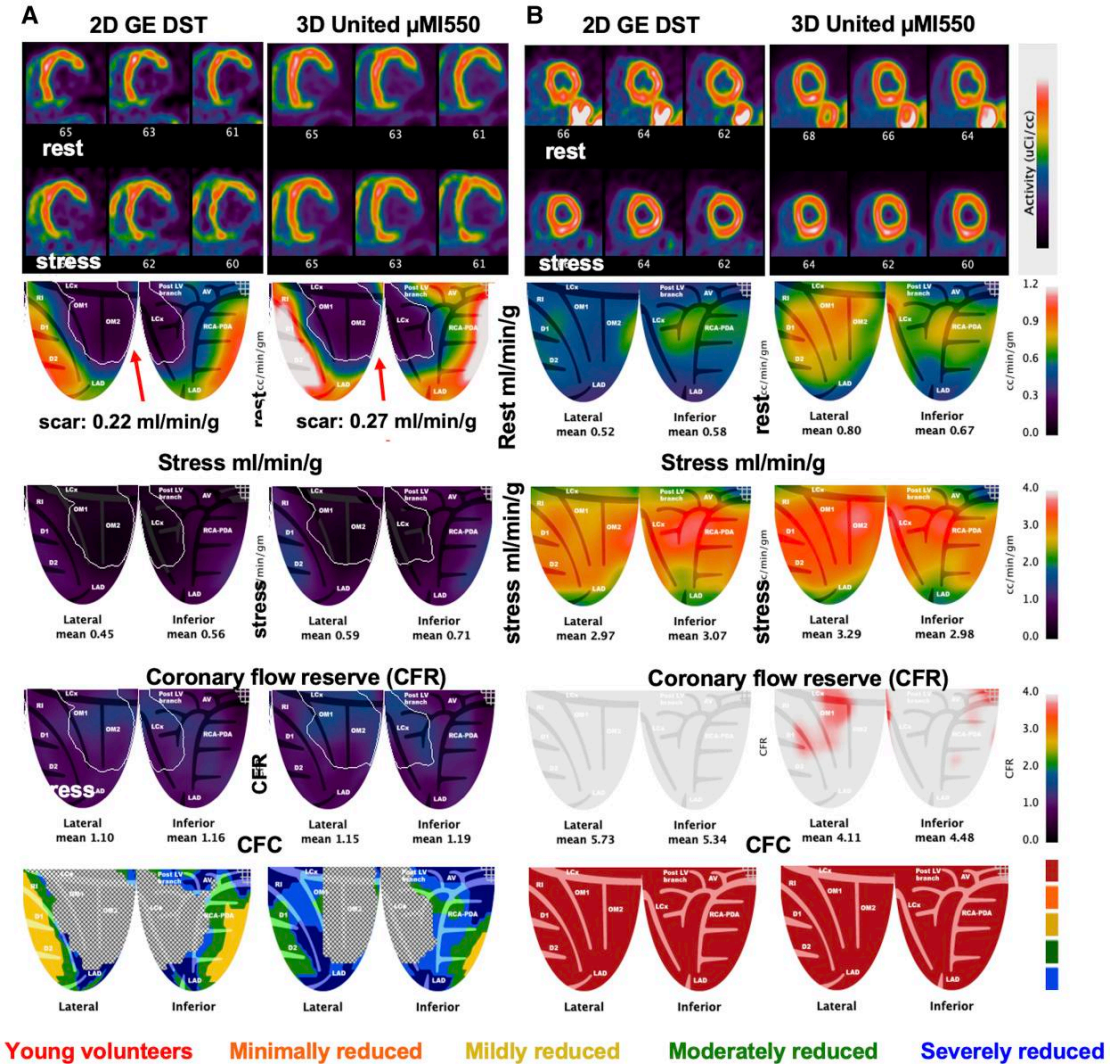
3D United µMI550 compared with established 2D GE DST for extremes of myocardial perfusion. (*A*) A 63-year-old male with known lateral-inferior myocardial infarction (MI), coronary artery bypass surgery (CABG), and PCI with reduced ejection fraction of 35%. Perfusion at rest in the outlined territory of transmural scar (white line) is 0.22 mL/min/g on the 2D DST and 0.27 on the United, respectively. (*B*) A 30-year-old male participant without risk factors or a family history of heart disease shows reproducibility between 2D and 3D PET-CT of rest and stress millilitres per minute per gram and CFR > 4 cc/min/g.

We compared 56 participants for ‘different-day’ rest–stress in millilitres per minute per gram measured by analogue 2D DST and the United over a wide spectrum of perfusion from myocardial scar to maximum stress perfusion. No bias was found between the two systems (paired *P* = 0.482) with similar precision (*P* = 0.321) and a COV of ±23% (*Table 3*). Separate rest and stress perfusion with CFR are compared in Supplementary data online, *Table S2*. *Figure 4* compares two pairs of cases at extreme ends of clinical severity. *Figure 4A* shows resting myocardial perfusion within automated iso-contour, software-tracked, lateral-inferior transmural scar for both scanners (outlined by a white line). Perfusion was comparable at 0.22 and 0.27 mL/min/g, respectively, consistent with the myocardial blood flow (MBF) range by cardiac PET for MRI-verified transmural scar.^10^  *Figure 4B* shows a 30-year-old healthy volunteer with comparably high stress perfusion over 3 mL/min/g and high CFR of 4.0–5.0.

### Biological plus methodological variability of different-day serial test–retest myocardial perfusion between two digital SiPM PET-CT scanners

**Figure 5:**
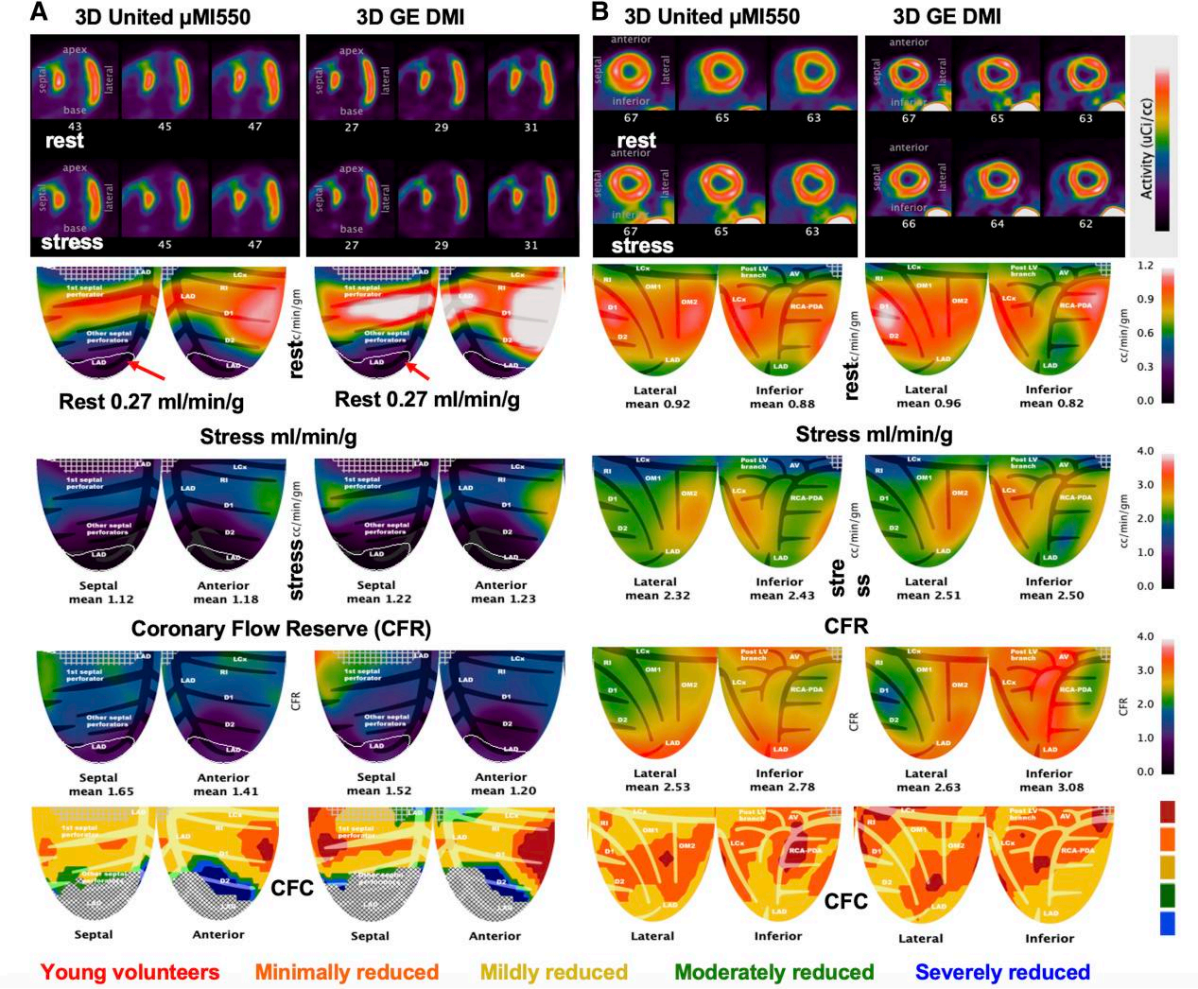
Serial images of two participants on different days on the 3D GE DMI and 3D United µMI550 for extremes of CAD severity. (*A*) The iso-contour of the fixed perfusion defect is identical at 0.27 mL/min/g for both scanners and within the acceptable range of MBF for transmural myocardial scar (17). (*B*) Participant with comparable perfusion, CFR, and CFC on each 3D system.

Reproducibility of PET rest–stress perfusion was also compared in the same fashion between United and DMI in 49 pairs. No bias was found between the two systems (paired *P* = 0.535) with similar precision (*P* = 0.467) and a COV of ±21% (*Table 3*). *Figure 5A* shows resting myocardial perfusion with apical transmural scar within the automated iso-contour, software-tracked, for both scanners (outlined by a white line). Perfusion was identical at 0.27 mL/min/g and within acceptable range of MBF for transmural scar.^10^  *Figure 5B* shows a clinical volunteer pair with comparable perfusion and CFR.

### Methodological and biological reproducibility of different-day and same-day serial test–retest myocardial perfusion for the 3D µMI550 SiPM PET-CT

Twenty-five participants underwent the expanded research protocols of *Figure 1* on the United to test for ‘different-day’ reproducibility. The ‘different-day’ perfusion measurements contain averaged rest from the rest–rest–stress protocol A to single rest measurement from B and vice versa for stress comparison. No significant difference was found for global ‘same-day’ or ‘different-day’ global perfusion (paired *P* = 0.053 and 0.181) with similar precision (*P* = 0.436 and 0.622) and a COV of ±11% and ±12% (*Table 3*). ‘Same-day’ quadrant flows show small bias in the anterior wall (*P* = 0.037) but no differences for ‘different-day’ quadrant measurements (see Supplementary data online, *Table S3*). While results of previous reports describe differences between ‘same-day’ and ‘different-day’ measurements, Supplementary data online, *Table S1* indicates United does not see those differences for short-term reproducibility (paired *P* = 0.349 and Pitman–Morgan *P* = 0.420).

### Methodological and biological reproducibility of different-day and same-day serial test–retest myocardial perfusion for the 3D DMI SiPM

The reproducibility parameters used for the United were also used for 24 participants on the 3D DMI. For the 3D DMI, the ‘different-day’ COV was ±17% (*Table 3*). ‘Same-day’ measurements of rest–stress perfusion were not significantly different, but ‘different-day’ measurements were significant (paired *P* = 0.213 and *P* = 0.028, respectively). Precision was similar between ‘same-day’ and ‘different-day’ (*P* = 0.492 and 0.334) (*Table 3*). ‘Same-day’ quadrant flows show no bias, but there was a significant difference for anterior and lateral walls for ‘different-day’ measurements (*P* = 0.040, *P* = 0.033) (see Supplementary data online, *Table S3*). Absolute differences were also significantly different (paired *P* = 0.003) but with similar precision (*P* = 0.193) (Supplementary data online, *Table S1*). Therefore, 3D DMI was slightly less accurate day-to-day than 3D µMI550, but within ranges of previously published COV (*Table 1*) and likely clinically insignificant. ‘Same-day’ and ‘different-day’ regional quadrant perfusion were comparable, with small significant bias for inferior and lateral walls, possibly due to cardiac motion (see Supplementary data online, *Table S3*).

### Summary composite methodological and biological reproducibility of ‘different-day’ serial test–retest myocardial perfusion in the same subject for the three PET-CT scanners

With similar bias, variance, and COV between subgroup scanner comparisons, all three systems were determined equivalent as the basis for combining all data. The *Graphical Abstract* summarizes all 154 paired ‘different-day’ perfusion comparisons of the three scanners: 2D vs. 3D, 3D vs. different 3D, and paired PETs on the same 3D scanner. The *Graphical Abstract A* shows an example of a participant with an unchanged relative stress defect over 13 years of follow-up for which reproducible quantitative perfusion is needed for establishing patient status. For this purpose, all data combined subgroup perfusion in millilitres per minute per gram are correlated in *Graphical Abstract B* with Bland–Altman plots in *Graphical Abstract C* of perfusion difference and relative difference (difference/mean) as further evidence of their similarity. Bland–Altman shows that most perfusion measurements fall within limits of agreement (LOA), where points beyond the LOA are well above the ischaemic threshold, with little clinical consequence. In *Graphical Abstract D*, the combination of all subgroups, shown in *Table 1*, produces the largest comparison to date of ‘different-day’ perfusion and maintains a similar mean difference of 0.028 ± 0.33 without a bias (paired *P* = 0.136) and a COV of ±20%.

**Figure 6:**
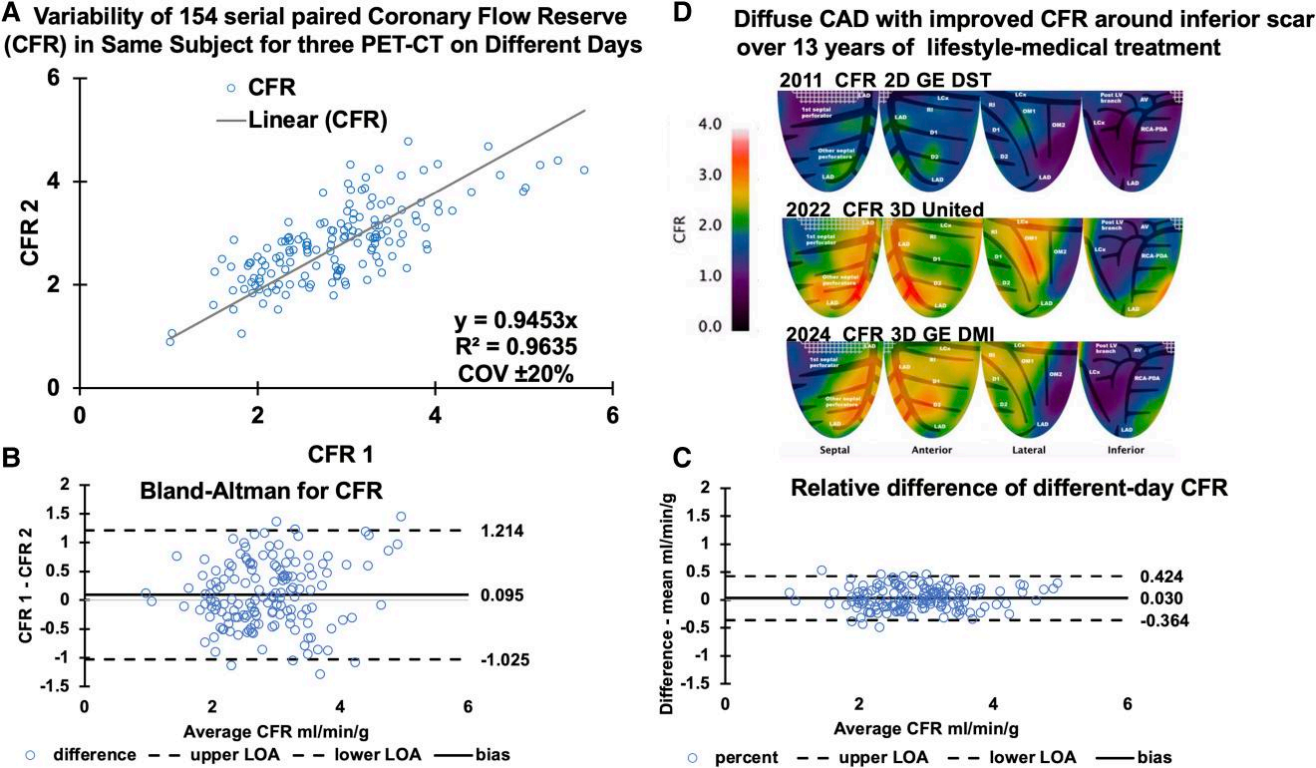
Day-to-different-day variability of 154 serial paired global CFR measurements among the analogue 2D GE DST, the 3D United µMI550, and the 3D GE DMI PET-CT. (*A*) Linear correlation of measurement one and measurement two. (*B*) Bland–Altman plots differences of serial CFR measurements. (*C*) Bland–Altman plot of relative percentage differences between serial CFR measurements. (*D*) Clinical example from *Graphical Abstract A* with severe, fixed, relative stress defect over 13 years, the CFR improved over the years of intense lifestyle-medical management without invasive procedures, illustrating the importance of reproducibility for personalized CAD management and randomized trials.

Since CFR is widely used for quantifying physiologic stenosis severity, *Figure 6* shows the corresponding summary for CFR in the same 154 paired PETs. The CFR COV was ±20% and comparable with the perfusion COV of ±20%, despite small bias (paired *P* = 0.041) driven by variability of rest flow from participants’ acclimatization to repeated measurements (*Figure 6*; Supplementary data online, *Table S2*). For patient with severe CAD and unchanged relative stress images over 13 years (*Graphical Abstract A*), the CFR maps in *Figure 6D* reveal improved CFR over years of intense lifestyle-medical management without invasive procedures.

**Figure 7:**
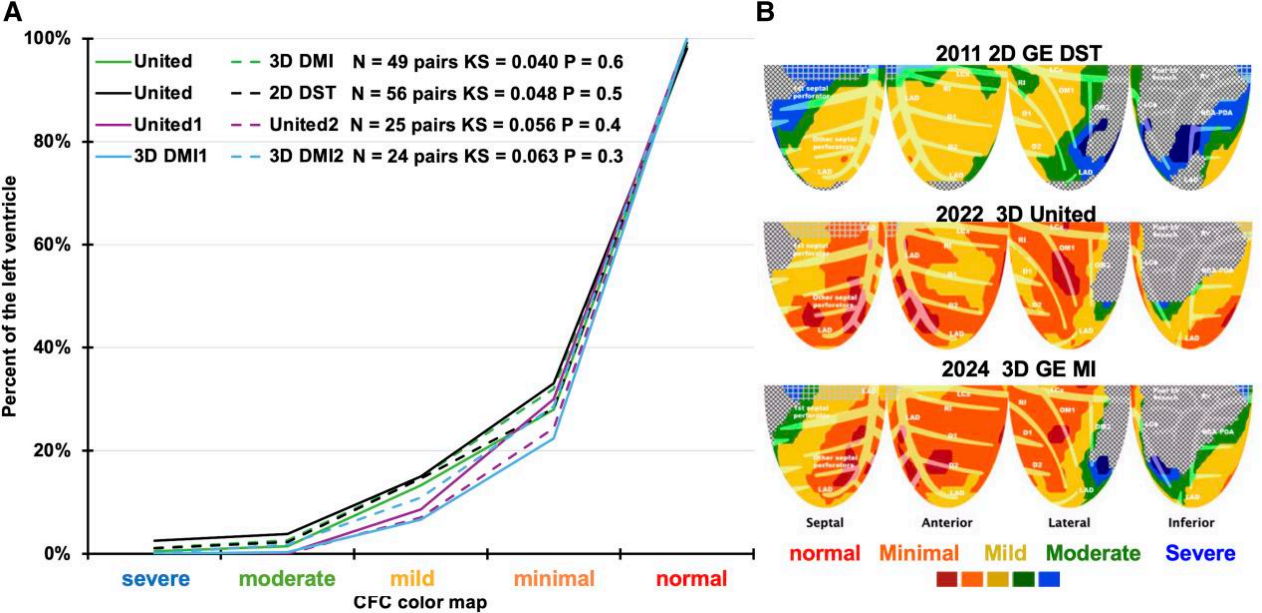
Reproducibility of 105 serial CFC maps of the same subject on different days for three different PET-CT by (*A*) the Kolmogorov–Smirnov test. 2D GE DST vs. 3D United µ550 and 3D United µ550 vs. 3D GE DMI. (*B*) Clinical example *Graphical Abstract A* with severe, fixed, relative stress defect over 13 years, the CFC improved over the years of intense lifestyle-medical management without invasive procedures.

The Kolmogorov–Smirnov tests and plots in *Figure 7A* show excellent reproducibility of the CFC maps for the 154 paired ‘different-day’ PETs from the three PET-CT systems. For the patient with severe CAD and unchanged relative stress images over 13 years (*Graphical Abstract A*), the CFC maps in *Figure 7B* reveal more comprehensively the improvement during the 13 years of intense lifestyle-medical management. There were no significant differences in any perfusion metric for earlier vs. later PET scans (see Supplementary data online, *Table S4*).

## Discussion

### Quantitative clinical coronary pathophysiology by PET-CT

An extensive literature using 2D PET-CT documents that comprehensive, non-invasive, absolute rest–stress millilitres per minute per gram, their ratio as absolute CFR, and their combination as CFC per pixel quantifies severe high mortality risk CAD that is reduced after non-randomized revascularization.^1–4^ Our results show that high-sensitivity 3D SiPM PET-CT has comparable reproducibility and precision equivalent to legacy 2D PET-CT systems, thereby extending the knowledge base from 2D to 3D PET-CT. However, 3D PET-CT requires more advanced acquisition and image reconstruction software, with essential scatter, randoms, and dead time corrections specific to 3D PET-CT.

The robustness of our perfusion per pixel model is reflected in zero (0%) failure of producing clinically comparable rest–stress myocardial perfusion, CFR, and CFC per pixel with 3D SiPM PET-CT compared with 20% failures for ‘digital’ 3D SiPM PET-CT using a different perfusion model^7^ vs. ‘analogue’ 3D PET-CT or for other models producing non-physiologic scar perfusion values.^10^ Reproducibility reported here can be confirmed for any 2D or 3D PET-CT acquisition protocol and perfusion model by a practical definitive two-part ‘gold standard test’. For MRI-proven transmural myocardial scar, experimental and clinical PET myocardial perfusion is ≤0.32 mL/min/g^10^ that our model achieves in contrast with resting scar perfusion averaging 0.42–0.85 mL/min/g with scatter ranging up to 2 mL/min/g with other perfusion models.^10^ For PET-CT on healthy young volunteers ≤ 40 years old with no risk factors (including no obesity), no recreational drugs, and no measurable blood caffeine,^20^ global rest–stress perfusion and CFR after dipyridamole stress will be 0.7 ± 0.15 and 2.55 ± 0.58 mL/min/g and 4.02 ± 0.85, respectively (*n* = 240).^13–15^

### Truth and quantitative tools

HeartSee is the scientific name for our unique, comprehensive, integrated, coronary pathophysiologic quantification based on 54 years of data driven by passion or compulsion to understand the truths about basic coronary pressure-flow pathophysiology and its transition to clinical cardiology, untainted by any financial conflict of interest. Robust clinical outcomes of CFC-guided interventions were derived from the physiologically driven flow model design that is fundamentally different from other flow models by its ‘perfusion per pixel’ that is essential for quantifying artery-specific size severity abnormalities of heterogeneous CAD.^1–4,7,13–21^ Statistical noise is reduced by integrating or averaging pixel activity over time after randoms, dead time, and scatter corrections.^12^ It contrasts to spatial averaging within large external ROIs encompassing adjacent arterial distributions of differing activities and flows of other models.

This basic design difference also allows for a ‘simple’ retention model^12^ and high-quality left atrial or aortic images for precise arterial input function,^14,16,17^ and measured scanner-specific partial volume corrections^19,21^ not feasible for other models. Objective, data-based, colour-coded severity thresholds are calibrated by angina or ST depression > 1 mm during stress imaging^14,15^ and related to mortality myocardial infarction (MI) or revascularization in large cohorts with and without revascularization over 14-year follow-up.^1–4,8,13–21^ Finally, the four view topographic maps of rest–stress perfusion, CFR, CFC, FFR, and relative subendocardial perfusion as seen fluoroscopically or at open-heart surgery summarize pathophysiology in familiar views for cardiologists or surgeons (*Figure 2*).

Clinical coronary pathophysiology to guide management of CAD evolved from basic experimental coronary stenosis for CFR, pharmacologic stress imaging, stenosis pressure-flow fluid dynamic equations,^22–25^ and subendocardial PET perfusion imaging that was awarded the 1978 von Hevesy Prize for Research in Nuclear Medicine.^26^ It was the basis for the senior author joining the University of Texas Medical School at Houston in 1979 to build the first dedicated cardiac PET scanner and imaging centre. In turn, it evolved to a recent randomized trial of comprehensive lifestyle-medical treatment with CFC by PET-CT to reserve coronary revascularization for severe CFC that significantly reduced interventions and death or MI compared with standard care in chronic CAD.^27^

### Study limitations

The study has some limitations. The number of participants is modest but larger than other imaging studies that could lead to a Type I or Type II error but minimized by consistency among multiple group comparisons. Data are from a single institution using one quantitative perfusion model software validated experimentally and clinically for outcomes providing consistency over time.

Our CFC per pixel model for artery-specific size severity of myocardial perfusion abnormalities may not be reproducible using other perfusion models. Stress with dipyridamole, adenosine, and dobutamine gives similar results.^18^ Regadenoson stress used per manufacturer instruction achieves only 80% of dipyridamole stress but improves to 90% when radionuclide is injected at 55 s after regadenoson injection. Our CFC per pixel model is adapted for N-13 ammonia with perfusion comparable with Rb-82.

Reproducibility and applicability of other PET-CT or perfusion models for guiding interventions require demonstrating comparable paired PET metrics in same patient across the spectrum of perfusion on comparably diverse PET-CT scanners, or mirroring our protocols, for stress millilitres per minute per gram, CFR, and CFC per pixel. Given several ‘CFC mimics’ like average global stress perfusion plus average global CFR rather than ‘true CFC per pixel’, we caution that ‘getting what we get requires doing what we do’, specifically CFC per pixel, not incomplete derived measurements called ‘CFC’ using different perfusion models, image acquisition protocols and technology requiring their own comparable validation.

## Conclusions

Quantitative rest–stress myocardial perfusion using high-count Rb-82 with the HeartSee model is reproducibly measured with equal accuracy and precision between 2D and 3D SiPM PET by paired serial PETs in the same person on different days with a COV of ±20% and for same day COV of ±14%. CFC by 2D or 3D SiPM PET-CT quantifies CAD severity, favouring lifestyle-medical treatment while directing coronary interventions to physiologically severe, high-risk, obstructive CAD having survival benefit after revascularization. Precision and accuracy of PET quantitative myocardial perfusion provide confidence to clinicians for personalized, physiologically guided management of CAD or randomized trials.

## Supplementary Material

qyae115_Supplementary_Data

## Data Availability

If accepted for publication, all data will be made available in xlsx files with its statistical analysis or in R code format on a University of Texas secure website or provided to individuals at specific written request of specific individuals for a specific purpose emailed or sent as hard copy to the first or senior author.

## Lead author biography

Amanda Elise Roby is the technical director of the Weatherhead PET Center, McGovern Medical School at UTHeath, Houston, Texas. She received her Bachelor of Science in Radiologic Sciences at the University of Oklahoma HSC as a Certified Nuclear Medicine Technologist (CNMT) in 2005 and her MBA in 2022. She served as a technologist in Arizona and transitioned to PET at UCSF and then MD Anderson, Houston, participating in clinical research projects and publications. In 2011, she became the lead technologist at the UT Weatherhead PET Center, progressing to her current position with energetic passion for patient care, technical knowledge, and self-taught statistical analysis.
