# Supplementary material for: Reproducibility of quantitative myocardial perfusion and coronary flow capacity by positron emission tomography: 3D digital silicon photomultiplier solid state vs. legacy 2D analogue systems for clinical practice and trials
Source: Eur Heart J Imaging Methods Pract. 2024 Dec 9;2(3):qyae115. doi: 10.1093/ehjimp/qyae115 (PMC11651154; doi:10.1093/ehjimp/qyae115)
Supplement: qyae115_Supplementary_Data [file qyae115_supplementary_data.docx]

**Supplement**  10-13-24


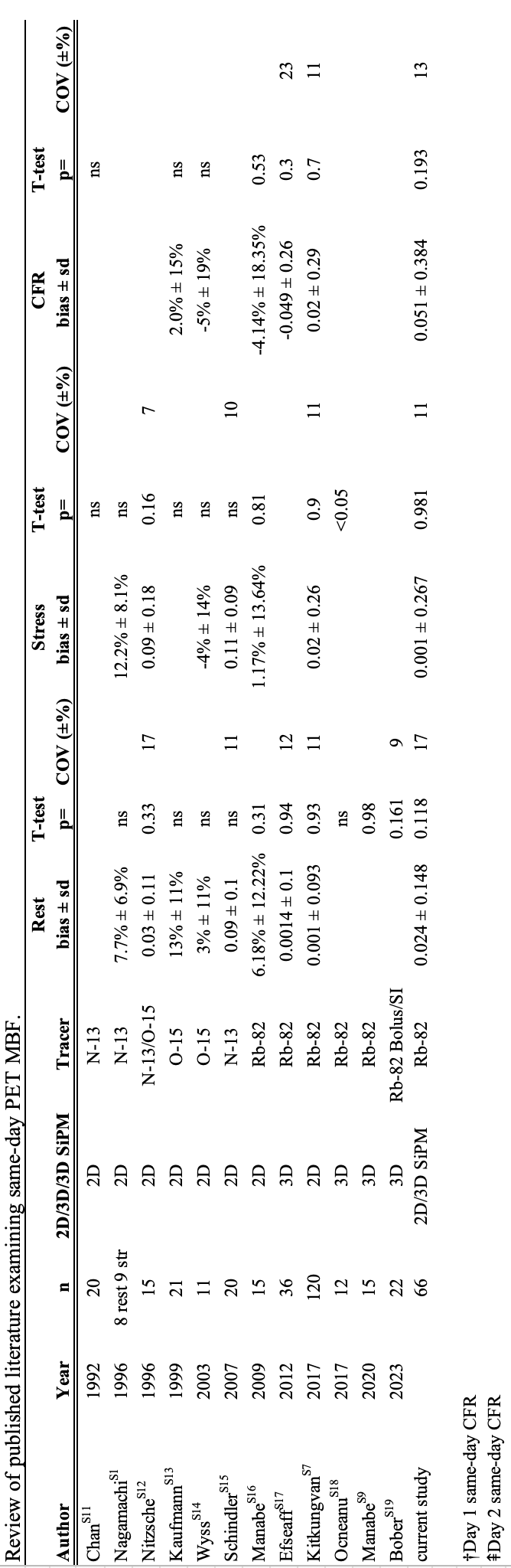


EHJ-IMP-D-24-00152 revised

**Supplement Table S0**

**Supplement Table S1**


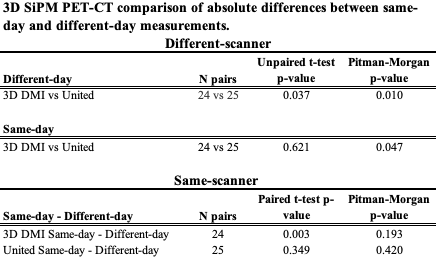


**Supplement Table S2**


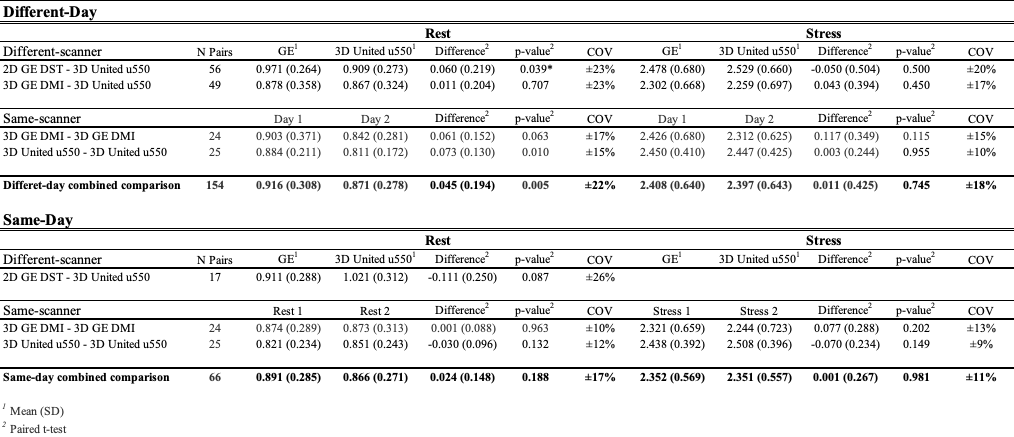

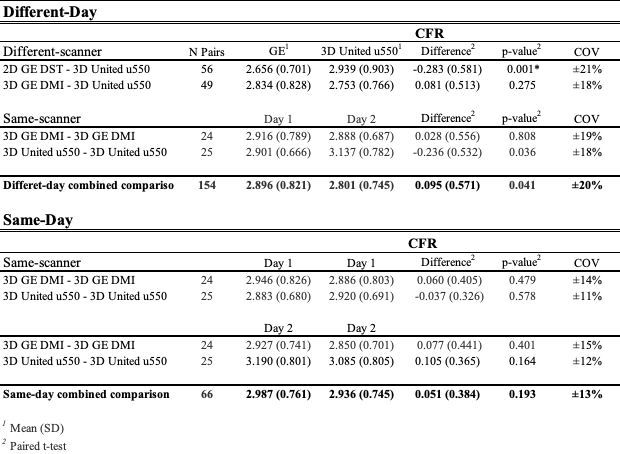


**Supplement Table S3**

**Supplement Table S4**

There were no significant differences in any perfusion metric for earlier versus later PET scans.

| **Time between paired flow measurements** | | | |
| --- | --- | --- | --- |
|  | **> 12 days^1^** | **< 12 days^1^** | **p-value^2^** |
|  | **N = 75** | **N = 79** |  |
| **Δ rest flow** | **0.01 (0.21)** | **-0.01 (0.19)** | **0.5** |
| **Δ stress flow** | **0.05 (0.45)** | **0.05 (0.36)** | **>0.9** |
| **Δ cfr** | **0.05 (0.60)** | **0.11 (0.56)** | **0.5** |
| **# days** | **20±10** | **7±2** | **<0.001** |

| **^1^ mean (sd)** |
| --- |
| **^2^ Welch two sample t-test** |

**Supplement Figure S1:** A) Correlation of the 3D United µMI550 to the 2D GE DST of *same-day* rest only MBF. B) Demographics of the 17 participants in the rest only sub-group. C) A simple linear regression model compares the difference in resting MBF (2D GE DST – 3D United) and participant BMI. The model is insignificant (r^2^ = 0.045; p = 0.206), suggesting BMI accounted for 4.5% of the variance in resting MBF between the scanners. The difference was positive for lower BMI, suggesting no scanner paralysis for the 3D United system without weight-based dosing.

**Supplement References for Table 1 main text**

References for Table 1 of main text.

1. Nagamachi S, Czernin J, Kim AS, Sun KT, Böttcher M, Phelps ME, Schelbert HR. Reproducibility of measurements of regional resting and hyperemic myocardial blood flow assessed with PET. J Nucl Med. 1996 Oct;37(10):1626-31. PMID: 8862296.
2. Jagathesan R, Kaufmann PA, Rosen SD, Rimoldi OE, Turkeimer F, Foale R, Camici PG. Assessment of the long-term reproducibility of baseline and dobutamine-induced myocardial blood flow in patients with stable coronary artery disease. J Nucl Med. 2005 Feb;46(2):212-9. PMID: 15695778.
3. Schindler TH, Zhang XL, Prior JO, Cadenas J, Dahlbom M, Sayre J, Schelbert HR. Assessment of intra- and interobserver reproducibility of rest and cold pressor test-stimulated myocardial blood flow with (13)N-ammonia and PET. Eur J Nucl Med Mol Imaging. 2007 Aug;34(8):1178-88. doi: 10.1007/s00259-007-0378-5. Epub 2007 Mar 3. PMID: 17334762.
4. Sdringola S, Johnson NP, Kirkeeide RL, Cid E, Gould KL. Impact of unexpected factors on quantitative myocardial perfusion and coronary flow reserve in young, asymptomatic volunteers. JACC Cardiovasc Imaging. 2011 Apr;4(4):402-12. doi: 10.1016/j.jcmg.2011.02.008. PMID: 21492816.
5. Johnson NP, Gould KL. Regadenoson versus dipyridamole hyperemia for cardiac PET imaging. JACC Cardiovasc Imaging. 2015 Apr;8(4):438-447. doi: 10.1016/j.jcmg.2014.11.016. Epub 2015 Mar 18. PMID: 25797122.
6. Kitkungvan D, Lai D, Zhu H, Roby AE, Johnson NP, Steptoe DD, Patel MB, Kirkeeide R, Gould KL. Optimal Adenosine Stress for Maximum Stress Perfusion, Coronary Flow Reserve, and Pixel Distribution of Coronary Flow Capacity by Kolmogorov-Smirnov Analysis. Circ Cardiovasc Imaging. 2017 Feb;10(2):e005650. doi: 10.1161/CIRCIMAGING.116.005650. PMID: 28213449.
7. Kitkungvan D, Johnson NP, Roby AE, Patel MB, Kirkeeide R, Gould KL. Routine Clinical Quantitative Rest Stress Myocardial Perfusion for Managing Coronary Artery Disease: Clinical Relevance of Test-Retest Variability. JACC Cardiovasc Imaging. 2017 May;10(5):565-577. doi: 10.1016/j.jcmg.2016.09.019. Epub 2016 Dec 21. PMID: 28017383.
8. Koenders SS, van Dalen JA, Jager PL, Knollema S, Timmer JR, Mouden M, Slump CH, van Dijk JD. Value of SiPM PET in myocardial perfusion imaging using Rubidium-82. J Nucl Cardiol. 2022 Feb;29(1):204-212. doi: 10.1007/s12350-020-02141-0. Epub 2020 May 14. PMID: 32410059; PMCID: PMC8873116.
9. Manabe O, Klein R, Katoh C, Magota K, deKemp RA, Naya M, Tamaki N, Yoshinaga K. Validation of regional myocardial blood flow quantification using three-dimensional PET with rubidium-82: repeatability and comparison with two-dimensional PET data acquisition. Nucl Med Commun. 2020 Aug;41(8):768-775. doi: 10.1097/MNM.0000000000001218. PMID: 32459715.
10. Byrne C, Kjaer A, Olsen NE, Forman JL, Hasbak P, Test–retest repeatability and software reproducibility of myocardial flow measurements using rest/adenosine stress Rubidium-82 PET/CT with and without motion correction in healthy young volunteers. J Nucl Cardiol. 2021 Dec; 28 (6):2860-2871. <https://doi.org/10.1007/s12350-020-02140-1>.
11. Chan SY, Brunken RC, Czernin J, Porenta G, Kuhle W, Krivokapich J, Phelps ME, Schelbert HR, Comparison of maximal myocardial blood flow during adenosine infusion with that of intravenous dipyridamole in normal men, JACC, 1992 Oct; 20 (4):979-985. https://doi.org/10.1016/0735-1097(92)90201-W.
12. Nitzsche EU, Choi Y, Czernin J, Hoh CK, Huang S, and Schelbert HR, Noninvasiv quantification of myocardial blood flow in humans. Circ, 1996 June; 93 (11):2000-2006. https://doi.org/10.1161/01.CIR.93.11.2000
13. Kaufmann PA, Gnecchi-Ruscone T, Yap JT, Rimoldi O, Camici PG. Assessment of the reproducibility of baseline and hyperemic myocardial blood flow measurements with 15O-labeled water and PET. J Nucl Med. 1999 Nov;40(11):1848-56. PMID: 10565780.
14. Wyss CA, Koepfli P, Mikolajczyk K, Burger C, von Schulthess GK, Kaufmann PA. Bicycle exercise stress in PET for assessment of coronary flow reserve: repeatability and comparison with adenosine stress. J Nucl Med. 2003 Feb;44(2):146-54. PMID: 12571202.
15. Schindler TH, Zhang XL, Prior JO, Cadenas J, Dahlbom M, Sayre J, Schelbert HR. Assessment of intra- and interobserver reproducibility of rest and cold pressor test-stimulated myocardial blood flow with (13)N-ammonia and PET. Eur J Nucl Med Mol Imaging. 2007 Aug;34(8):1178-88. doi: 10.1007/s00259-007-0378-5. Epub 2007 Mar 3. PMID: 17334762.
16. Manabe O, Yoshinaga K, Katoh C, Naya M, deKemp RA, Tamaki N. Repeatability of rest and hyperemic myocardial blood flow measurements with 82Rb dynamic PET. J Nucl Med. 2009 Jan;50(1):68-71. doi: 10.2967/jnumed.108.055673. Epub 2008 Dec 17. PMID: 19091892.
17. Efseaff M, Klein R, Ziadi MC, Beanlands RS, deKemp RA. Short-term repeatability of resting myocardial blood flow measurements using rubidium-82 PET imaging. J Nucl Cardiol. 2012 Oct;19(5):997-1006. doi: 10.1007/s12350-012-9600-3. Epub 2012 Jul 24. PMID: 22826134.
18. Ocneanu AF, deKemp RA, Renaud JM, Adler A, Beanlands RS, Klein R. Optimally Repeatable Kinetic Model Variant for Myocardial Blood Flow Measurements with ^82^Rb PET. Comput Math Methods Med. 2017;2017:6810626. doi: 10.1155/2017/6810626. Epub 2017 Feb 13. PMID: 28293274; PMCID: PMC5331165.
19. Bober RM, Milani RV, Kachur SM, Morin DP. Assessment of resting myocardial blood flow in regions of known transmural scar to confirm accuracy and precision of 3D cardiac positron emission tomography. EJNMMI Res. 2023 Sep 27;13(1):87. doi: 10.1186/s13550-023-01037-7. PMID: 37752344; PMCID: PMC10522549.
